# Multi-b-values-fitting readout-segmentation of long variable echo-trains diffusion-weighted imaging (RESOLVE DWI) in evaluation of disease activity and curative effect of axial spondyloarthritis (axSpA)

**DOI:** 10.3389/fimmu.2023.1136925

**Published:** 2023-07-03

**Authors:** Xianyuan Chen, Shengsheng Yang, Mingui Lin, Fei Gao, Mingping Ma, Shun Yu

**Affiliations:** ^1^ Shengli Clinical Medical College of Fujian Medical University, Fujian, Fuzhou, China; ^2^ Department of Radiology, Fujian Provincial Hospital, Fujian, Fuzhou, China; ^3^ Department of Radiology, Fuzhou Second Hospital, Fujian, Fuzhou, China; ^4^ Department of Rheumatism, Fujian Provincial Hospital, Fujian, Fuzhou, China

**Keywords:** RESOLVE DWI, multi-b-values-fitting, axial spondyloarthritis, quantitative, tumour necrosis factor inhibitors

## Abstract

**Background:**

Disease activity is relevant to the treatment and prognosis of axSpA, and methods to quantitatively assess disease activity and efficacy of axSpA are still being explored.

**Objective:**

The purpose of this study was to find an optimal quantitative indicator for evaluating disease activity and curative effect of axSpA, using multi-b-values-fitting RESOLVE DWI.

**Methods:**

The prospective study included 106 patients divided into axSpA group (n=89) and no-axSpA group (n=17) by Assessment of Spondyloarthritis international Society (ASAS) criteria. The axSpA group were divided into active group and inactive group according to ASDAS-CRP. The active group treated with systematic tumour necrosis factor inhibitors (TNFi) was selected as treatment group (n=20). All patients underwent MRI examination of sacroiliac joints (SIJs), including RESOLVE DWI. The ADC values of subchondral bone marrow in SIJs were measured (ADC_50,500_ was b=50,500s/mm^2^ fitting, ADC_50,700_ was b=50,700s/mm^2^ fitting, and ADC_50,500,700_ was b=50,500,700s/mm^2^ fitting). By comparing the ADC values between different groups, a relatively optimal b-values-fitting sequence was obtained, further evaluating curative effect of the treatment group.

**Resultd:**

The ADC values of axSpA group, inactive group and active group SIJs were all higher than those of no-axSpA group. The ADC values of active group SIJs were all higher than those of inactive group. ADC_50,500,700_ had the largest AUC, relative higher sensitivity and specificity while taking account of the image quality than ADC_50,700_ and ADC_50,500_ between different groups. In the treatment group, there was no significant difference in ADC values between pre-treatment and 3 weeks, 3 weeks and 6 weeks, 6 weeks and 12 weeks (all P>0.0083, Bonferroni-corrected threshold), while the decreased ADC values in the interval of 6 weeks or more were statistically significant (all P<0.0083, Bonferroni-corrected threshold).

**Conclusion:**

Multi-b-values-fitting (b=50,500,700s/mm^2^) RESOLVE DWI has a certain advantage in evaluating disease activity of axSpA. It was worth noting that short-term review (3 weeks or less) of RESOLVE DWI was unsatisfactory and review at 6 weeks or later would help to evaluate curative effect of axSpA.

## Introduction

AxSpA is a chronic systemic autoimmune disease involving sacroiliac joints, predominantly affecting sacroiliac joints and correlated with HLA-B27 (1, 2). The main symptom is inflammatory low back pain (LBP). By virtue of its long course, poor prognosis and high disability rate, it seriously affects the quality of life and working ability of patients (3, 4). Early diagnosis and early treatment are particularly important.

The study about the pathogenesis of sacroiliitis displayed that pannus formation in subchondral tissue may be the pathological sign of sacroiliac arthritis in the early stage, followed by inflammatory cell infiltration, pannus formation progress bone plate injury and cartilage invasion (5). In addition, several inflammatory factors, including TNF- α, were also involved in the above-mentioned inflammatory response process, which were also targets for clinical drug therapy (6, 7). Therefore, the inflammatory response, caused by inflammatory cells and factors, play a vital role in the occurrence and development of sacroiliac arthritis. The process of inflammatory reaction caused by inflammatory cells can be characterized by the increase of laboratory indicators (CRP, ESR) and the formation of local bone marrow edema (BME). In 2009, ASAS proposed that imaging changes of active inflammation of sacroiliac joints play an important role in the diagnosis of axSpA, especially MRI (8). In 2019, ASAS updated consensus definitions for MRI lesions of the sacroiliac joints (9). By virtue of multiple sequences and directions, MRI can more sensitively distinguish the signal of BME of sacroiliac joints (10).

Conventional DWI is a functional MRI technique, which can non-invasively evaluate the random motion of water molecules by measuring ADC values. In several studies, ADC values as a quantitative indicator were used to diagnose SpA and evaluate the disease activity of patients with axSpA (11–15). However, conventional DWI is vulnerable to geometric distortion, T2*-induced blurring, and susceptibility artifacts, which will reduce the quality of the image and the detection of lesions. RESOLVE-DWI has been suggested as an alternative approach to overcome the limitations of conventional DWI through innovative technology, whose core is that the k-space is divided into several segments along the direction of the readout (16–18). The superiority of the RESOLVE DWI sequence has been displayed in various organs, including the brain, head and neck, and pelvis (19–22), except the axial skeleton. Furthermore, in the study of Salma et al., the fitting of multiple low b-values can achieve similar lesion detection rates with high b-values (23). There were few reports about the application of RESOLVE DWI in the sacroiliac joints, and even fewer with multiple b-values fitting.

The clinical treatment decision of the patients with axSpA depends on the disease activity, evaluated by ASDAS widely, which is a comprehensive assessment of clinical symptoms and laboratory data (24). At the present time, reducing disease activity by comprehensive treatment, including TNFi (25, 26), is recommended for patients with high activity (ASDAS≥2.1) (27). ASAS/EULAR, jointly developed by axSpA guidelines, recommended in 2016 that patients with continuous relief of inflammation should be considered for reducing TNFi (28), which was help for timely adjustment of clinical treatment plan to reduce the financial burden of patients and side effects. Currently, although the ASDAS score is widely accepted in assessing disease activity of axSpA and clinical monitoring the curative effect of axSpA, it is affected by subjective factors of the patients, which may cause some bias.

In this study, multi-b-values fitting RESOLVE DWI was used in the examination of sacroiliac joints in patients with axSpA, no-axSpA or treatment to discuss the value of multi-b-values-fitting RESOLVE DWI in distinguishing the inflammatory activity of sacroiliac joints and evaluate and monitor curative effect of axSpA.

## Materials and methods

### Participants

This prospective clinical study was conducted with the approval of the institutional review board (K2016-04-015, K2020-07-023). Written informed consent was obtained from all of the participants.

This study collected 89 participants with axSpA (axSpA group) and 17 participants with normal sacroiliac joints (no-axSpA group), admitted to our institution from May 2017 to May 2021 (**Supplementary Figure S1**). A total of 89 individuals met the inclusion criteria of axSpA group: accordance with the ASAS (8), low back pain persisted for more than 3 months, age at symptom onset of less than 45 years. A total of 17 individuals met the inclusion criteria of no-axSpA group: sacroiliac joints MR examination because of simple chronic low back pain, absence of high signal intensity in subchondral bone marrow of bilateral sacroiliac joints in fat suppression proton density weighted imaging (PDWI), exclusion from axSpA. Exclusion criteria: contraindications of MR examination, primary osteoporosis, immune system diseases, tumors, axSpA complicated with other bone diseases or history of using hormones and immune drugs in recent 6 months, incomplete data, including clinical, laboratory and MRI. In addition, the activity group treated with systematic TNFi (Etanercept, manufacturer: Pfizer Investment Co., Ltd.; related precautions were operated according to the instructions; usage: once a week, once 50mg.) was selected as the treatment group(n=20). The treatment group began regular TNFi treatment after the first MR examination.

### Experimental grouping

Two rheumatologists applied the ASAS classification criteria to classify participants into either the axSpA group or the no-axSpA group, independently. Disagreement between the rheumatologists was resolved in consensus. For a total of 89 participants diagnosed with axSpA according to the criteria of ASAS (54 male and 35 female participants; age: median, 32.0 years; IQR: 25.0–47.5 years; range, 17–71 years). The remaining 17 participants were classified as no-axSpA group (10 male and 7 female participants; age: median, 39.0 years; IQR, 29.5–45.0 years; range, 17–57 years) (**Table 1**). ASDAS which assesses the activity of ax-SpA is associated with back pain, morning stiffness, patient global assessment, peripheral pain, swelling, and CRP level, as detailed in the formula in **Table 1**. The axSpA group with ASDAS <1.3 was divided into the inactive group and the ASDAS≥1.3 was divided into the active group. There was no significant difference in sex and age among no-axSpA group, axSpA group, active group and inactive group (all P>0.05) (**Supplementary Table S1**).

**Table 1:** Demographic and Clinical Characteristics.

	no-axSpA (n = 17)	axSpA (n = 89)	axSpA(n = 89)		Treatment group^*^			
			Inactive (n = 29)	Active (n = 60)	Pre-treatment (n=20)	3 weeks (n=20)	6 weeks (n=20)	12 weeks (n=20)
Age (y)	39.0(29.5,45.0)	32.0(25.0,47.5)	32.0(25.0,41.0)	32.0(24.3,48.0)	26.5(21.3,32.0)	—	—	—
Sex								
Male	10	54	17	37	11	—	—	—
Female	7	35	12	23	9	—	—	—
HLA-B27	0	74	24	50	17	—	—	—
CRP	2.00(2.00,2.00)	22.19(4.16,59.78)	2.80(2.00,4.16)	47.70(20.51,72.76)	49.19(38.98,61.21)	11.12(3.51,19.81)	4.11(2.54,5.14)	2.00(2.00,2.00)
ASDAS-CRP	0.60(0.60,0.60)	2.00(1.10,3.25)	0.80(0.60,1.10)	2.90(2.00,3.60)	2.90(2.63,3.63)	1.60(0.88,1.98)	0.90(0.80,1.10)	0.60(0.60,0.75)

IQR, interquartile range; HLA-B27, human leukocyte antigen-B27; CRP, C-reactive protein; ASDAS-CRP, Ankylosing Spondylitis Disease Activity Score–C-reactive protein.

ASDAS-CRP: ASDAS-CRP = [0.121 × back pain + 0.058 × duration of morning stiffness + 0.110 × patient global assessment + 0.073 × peripheral pain and swelling + 0.579 × ln (CRP + 1)]. When the conventional CRP level is below the limit of detection or when the hsCRP level is <2 mg/liter, the constant value of 2 mg/liter should be used to calculate the ASDAS-CRP score.

*, selected from the active group.

### MRI techniques and image acquisition

The MRI examinations were performed on a 1.5T unit (MAGNETOM Aera, Manufacturer: Siemens Magnetic Resonance Co., Ltd.), using the body flexed array coil in the supine position participants. All participants’ head entered firstly, and the transverse and oblique coronal positions were scanned from the upper edge of the pelvis to the acetabulum. The scanning sequences included conventional transverse axial T1WI, T2WI and T2WI-fs, oblique coronal T1WI, fat suppression proton density weighted imaging (PDWI), and RESOLVE DWI (**Table 2**).

**Table 2:** Conventional sacroiliac joint MRI sequences and imaging parameters.

b value (s/mm^2^)	T1WI	T2WI	T2WI-fs	fs-PDWI	RESOLVE DWI		
					50,500	50,700	50,500,700
Imaging	Coronal oblique	axial oblique	axial oblique	Coronal oblique	Coronal oblique	Coronal oblique	Coronal oblique
TR (ms)	582	3200	4000	3880	4760	4830	4890
TE (ms)	7.4	68	57	43	78138	80140	82144
FOV (mm)	240	220	220	240	240	240	240
Slice thickness (mm)	3	4	4	3	3	3	3
Flip angle	150	150	150	150	180	180	180
No. of slice	18	20	20	18	18	18	18
Gap(mm)	0.6	0.8	0.8	0.6	0.6	0.6	0.6
Matrix	256×320	256×320	205×256	205×256	100×100	100×100	100×100
Echo spacing(ms)	7.44	8.56	8.2	8.56	0.34	0.34	0.34
Bandwidth (Hz/pixel)	223	193	193	171	641	641	641
Parallel imaging	GRAPPA 2	GRAPPA 2	GRAPPA 2	GRAPPA 2	NONE	NONE	NONE
Fat suppression	NONE	SPAIR	NONE	SPAIR	SPAIR	SPAIR	SPAIR
Acquisition time (min:s)	1:41	1:04	2:00	2:04	6:38	6:41	8:58

**Figure 1:**
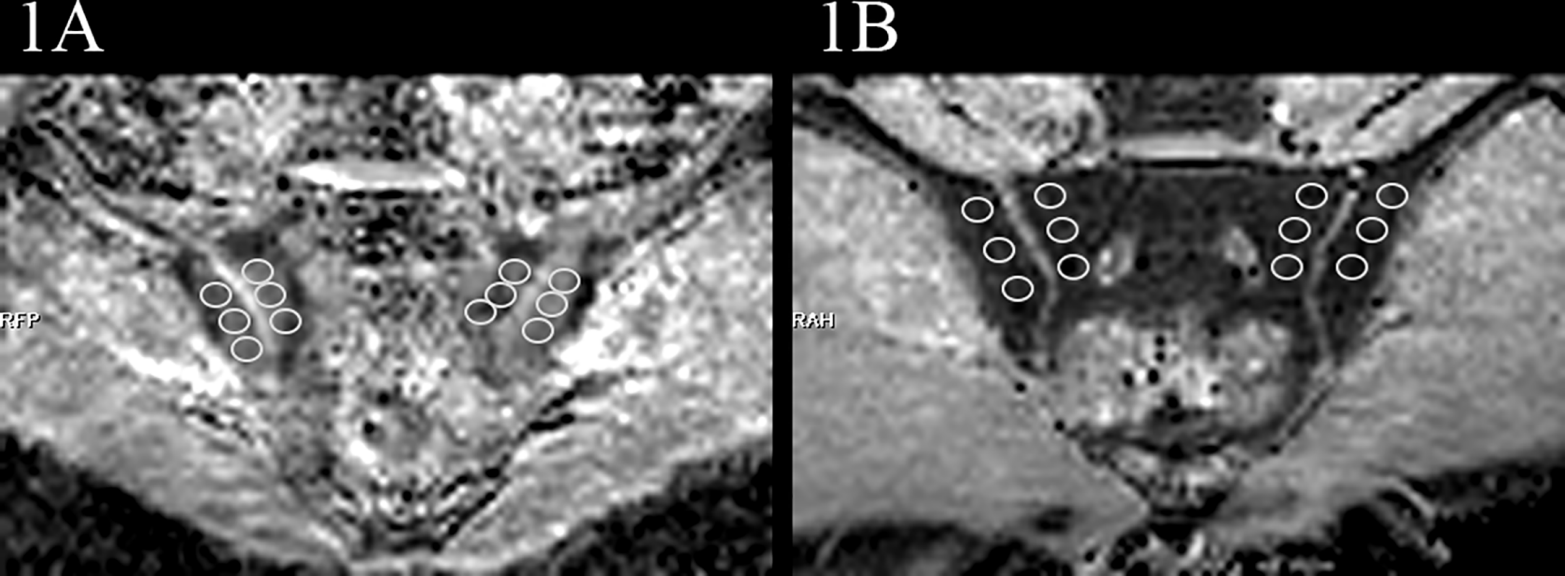
Examples of ROIs placement in the ADC maps (b=50,500,700s/mm^2^). **(A)** images of the participant with BME showed ROIs. **(B)** images of the participant without BME showed ROIs.

At the end of the study period, all MRI examination images (routine MRI sequences and RESOLVE DWI) of the participants were evaluated by two musculoskeletal radiologists, who were blinded to the clinical, laboratory and imaging data. Disagreement between the radiologists was resolved in consensus. The interpreters manually placed ROIs on the corresponding areas of the sacroiliac joints on ADC maps, where the ROI neared but not included the articular cartilage and avoided bone cortex and blood vessels. Referring to conventional MRI sequences, the sacroiliac joints were divided into four subchondral portions (left ilium, left sacrum, right ilium, and right sacrum), and three ROIs were placed on each subchondral portion. To guide ROI placement, the interpreters assessed the sacroiliac joints and took BME as a marker of active inflammatory changes. BME was deemed to be present if the reader identified hyperintense area on PDWI-fs. If BME was detected in the subchondral bone, three non-overlapping ROIs (25–35 mm^2^) (**Figure 1A**) were placed on areas of the ADC maps corresponding with the highest signal intensity on the PDWI-fs. If BME was not detected, then three non-overlapping ROIs (25–35 mm^2^) (**Figure 1B**) were placed on superior, middle, and inferior subchondral bone marrow. Through this process, 12 ROIs were obtained from the ADC maps each participant. The mean ADC values of the 6 ROIs on the sacral or iliac sides were taken as the final values. The musculoskeletal radiologists with 6 years of experience performed manual image measurements twice with an interval of at least 3 months to assess intra-observer agreement.

### Statistical analysis

All statistical analyses were performed with the Statistical Package for the Social Sciences (SPSS, version 25.0, Inc., Chicago, IL, USA). Categorical variables are presented as frequencies. The Shapiro-Wilk tests were conducted to analyses normality on the continuous variables. Data following normal distribution was represented by means ± standard deviation (SD) and non-normally distributed data was represented by median and IQR. The Wilcoxon test was performed to compare ADC values of sacral or iliac sides in sacroiliac joints within each group. The Kruskal-Wallis test was used to compare the difference of ADC values between each group. The receiver operating characteristic (ROC) curve was used to evaluate the diagnostic efficiency of ADC values obtained by fitting with different b values between each group, by calculating area under the curve (AUC), sensitivity, specificity, cut-off value and Youden index. Friedman test was used to compare the ADC values and ASDAS-CRP before treatment, 3 weeks, 6 weeks and 12 weeks after treatment. The Bonferroni correction for multiple comparisons was applied for the comparison of ADC values among the six possible pairwise combinations of different treatment cycles, and P’ <0.0083 showed statistical significance. For the remaining statistical analyses, P< 0.05 was considered to be statistically significant.

## Results

### Routine MRI findings of participants in each group

**Figure 2:**
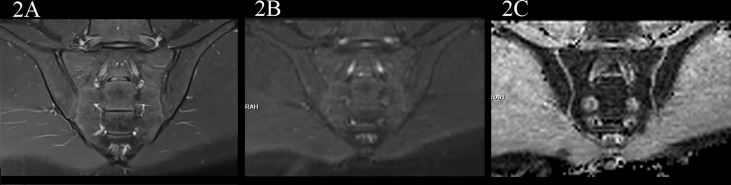
male, 24 years old, HLA-B27: -, CRP:<0.715mg/L, ASDAS-CRP:0.6, belonged to the no-axSpA group. **(A, B)** The coronal plane of PDWI-fs and RESOLVE DWI (b=50,500,700s/mm^2^) showed no obvious high signal intensity in the subchondral bone of bilateral sacroiliac joints, and the corresponding articular surfaces are smooth. **(C)** The ADC maps (b=50,500,700s/mm^2^) showed that the ADC values of subchondral bone marrow of bilateral sacroiliac joints were 0.455×10^-3^ mm^2^/s (iliac) and 0.487×10^-3^ mm^2^/s (sacral), respectively.

**Figure 3:**
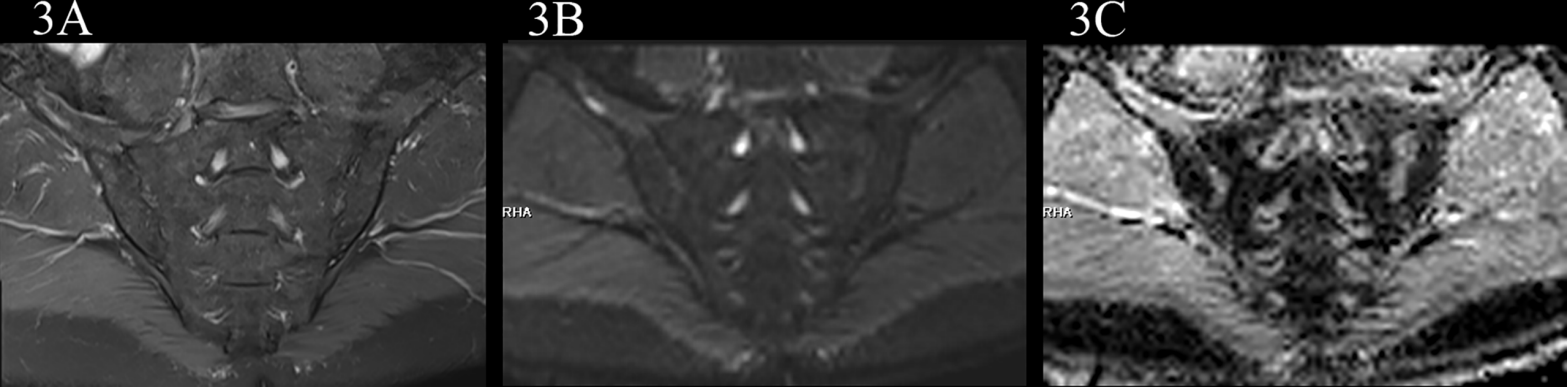
male, 61 years old, HLA-B27: +, CRP:2.85mg/L ASDAS-CRP:0.9, belonged to the inactive group. **(A, B)** The coronal plane of PDWI-fs and RESOLVE DWI (b=50,500,700s/mm2) did not show significant high signal intensity in subchondral bone marrow area of the sacroiliac joints and slightly rough bilateral sacroiliac joints surfaces. **(C)** The ADC maps (b=50,500,700s/mm^2^) showed the ADC values of subchondral bone marrow in bilateral sacroiliac joints were 0.593×10^-3^ mm^2^/s (iliac) and 0.723×10^-3^ mm^2^/s (sacral), respectively.

**Figure 4:**
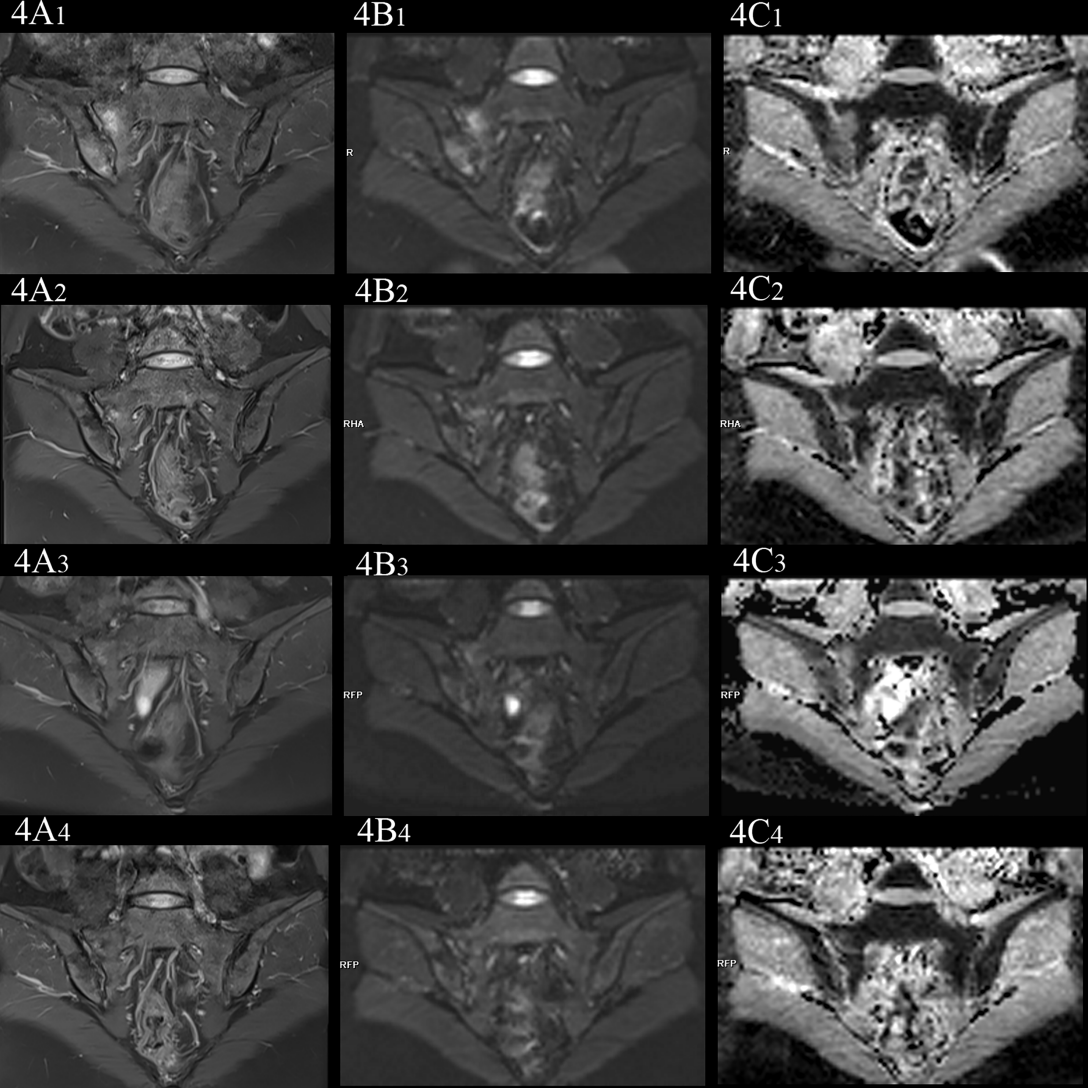
female, 33 years old, HLA-B27: +, CRP:21.7mg/L, ASDAS-CRP:3.4, belonged to active group and the treatment group; **(A_(1-4)_-C_(1-4)_)** showed the same patient before treatment and 3 weeks, 6 weeks and 12 weeks after treatment, respectively. **(A_(1-4)_)** showed the coronal plane of PDWI-fs; **(B_(1-4)_)** showed the coronal plane of RESOLVE DWI (b=50,500,700s/mm^2^); **(C_(1-4)_)** showed the ADC maps (b=50,500,700s/mm^2^). In patients with effective treatment, the ASDAS-CRP score decreased with the increase of the treatment cycle (3.4, 0.6, 0.6, 0.6). **(A_(1-4)_, B_(1-4)_)** PDWI-fs and RESLOVE DWI showed that the signal in the subchondral bone of bilateral sacroiliac joints decreases, gradually; **(C_(1-4)_)** showed that the ADC values in the bone marrow area of the lesion decreased gradually (1.483, 1.327, 1.027, 0.831×10^-3^ mm^2^/s).

In the no-axSpA group, there was no obvious abnormality in sacroiliac joints, the joint spaces on both sides were normal, the joint surfaces were smooth, and the bone signals were uniform. PDWI-fs showed no high signal (**Figure 2A**), DWI showed slightly low signal (**Figure 2B**), and ADC maps showed low signal (**Figure 2C**). The MRI findings of sacroiliac joints in axSpA group mainly included BME, fat deposition and bone erosion. The lesions of BME showed low signal on T1WI, high signal on PDWI-fs, high, equal and slightly high signal on RESOLVE DWI, and high signal on ADC maps. In the inactive group, the high signal intensity of bilateral sacroiliac joints was not obvious on PDWI-fs, RESOLVE DWI and ADC maps (**Figures 3A–C**). With the aggravation of BME, the higher signals on PDWI-fs (**Figures 3A**, **4A**_1_ and **5A**_1_), the higher signals on DWI (**Figure 3B**, **4B**_1_ and **5B**_1_) and ADC maps (**Figure 3C**, **4C**_1_ and **5C**_1_).

**Figure 5:**
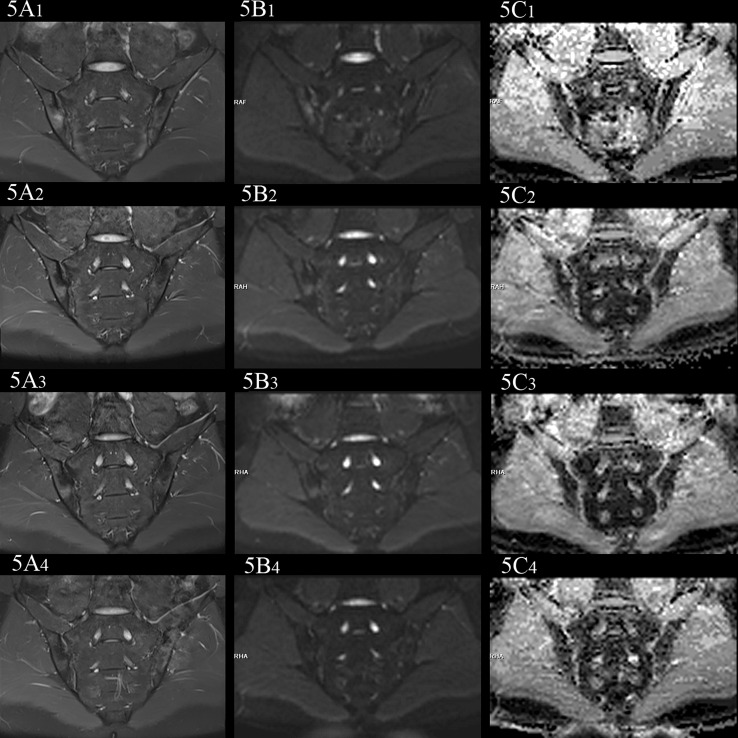
female, 27 years old, HLA-B27: +, CRP:31.5mg/L, ASDAS-CRP:4.6, belonged to active group and the treatment group; **(A_(1-4)_-C_(1-4)_)** showed the same patient before treatment and 3 weeks, 6 weeks and 12 weeks after treatment, respectively. **(A_(1-4)_)** showed the coronal plane of PDWI-fs; **(B_(1-4)_)** showed the coronal plane of RESOLVE DWI (b=50,500,700s/mm^2^); **(C_(1-4)_)** showed the ADC maps (b=50,500,700s/mm^2^). In patients with effective treatment, the ASDAS-CRP score decreases with the increase of the treatment cycle (4.6, 3.5, 2.2, 1.5). **(A_(1-4)_, B_(1-4)_)** PDWI-fs and RESLOVE DWI showed the gradual decrease of the signal in the subchondral bone of bilateral sacroiliac joints; **(C_(1-4)_)** showed the ADC values in the bone marrow area of the lesion decreased gradually (1.047, 0.710, 0.666, 0.648×10^-3^ mm^2^/s).

### Comparison of different ADC values of sacral or iliac sides in sacroiliac joints within each group

**Figure 6:**
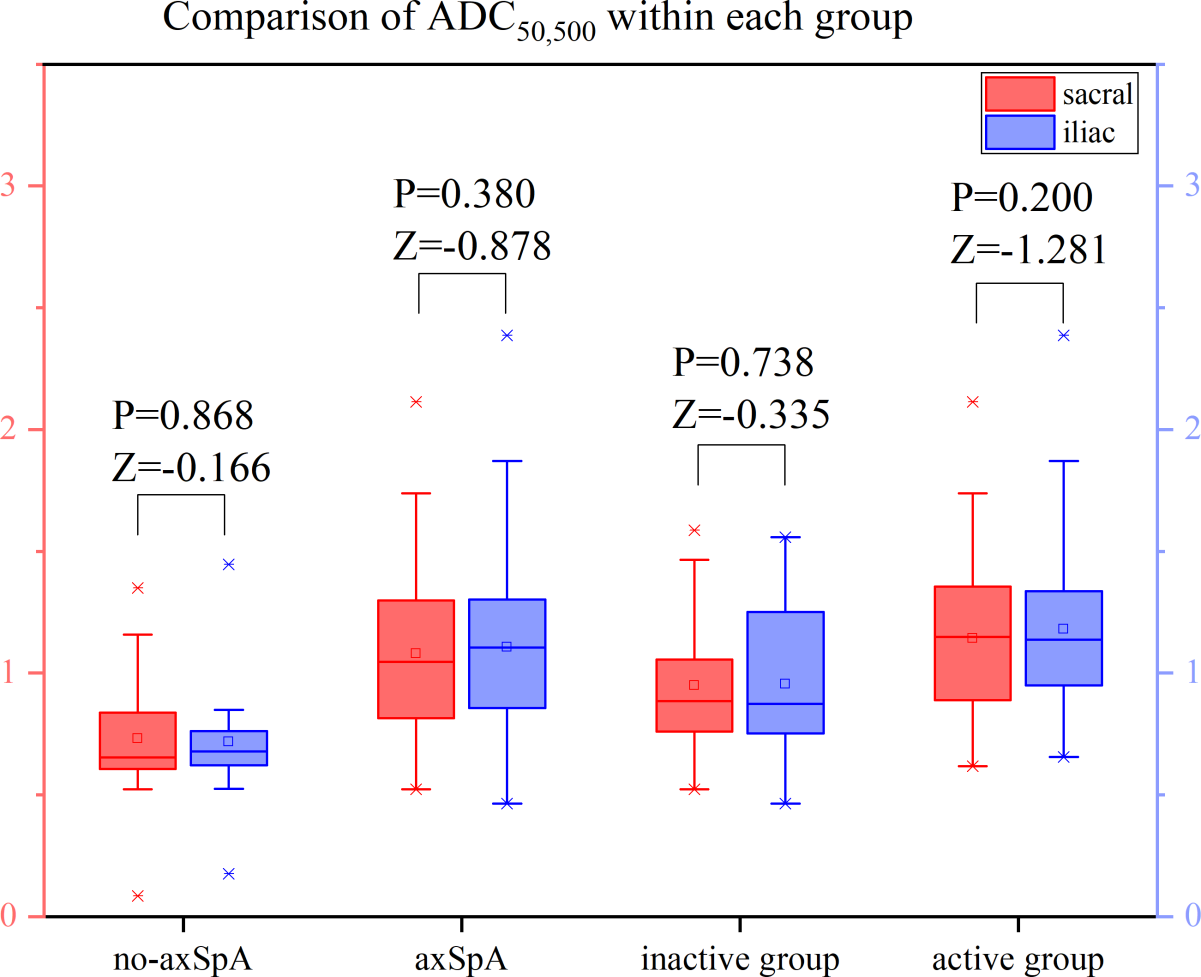
Boxplot for Comparison of ADC_50,500_ within each group. p-value of <0.05 was considered statistically signifificant.

**Figure 7:**
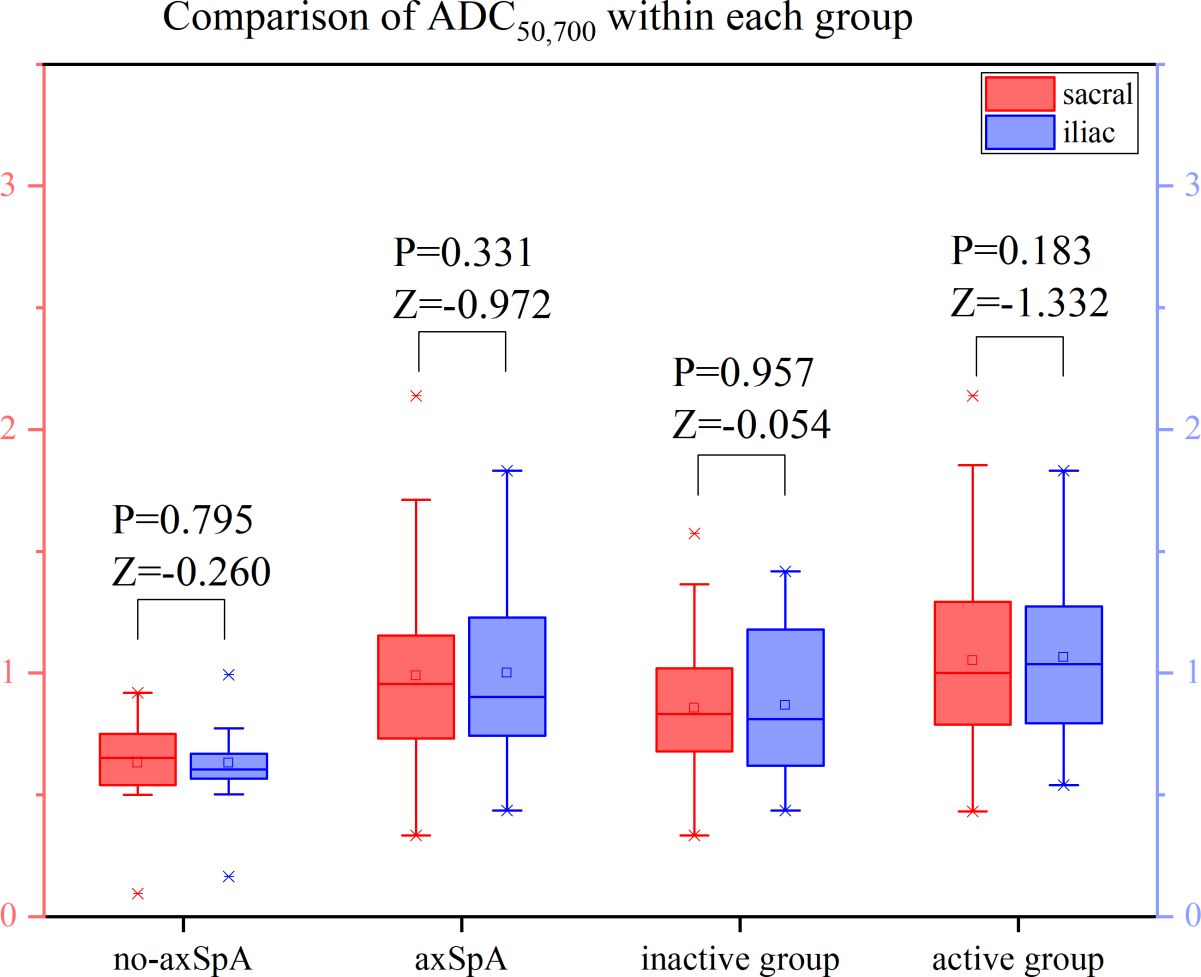
Boxplot for Comparison of ADC_50,700_ within each group. p-value of <0.05 was considered statistically signifificant.

**Figure 8:**
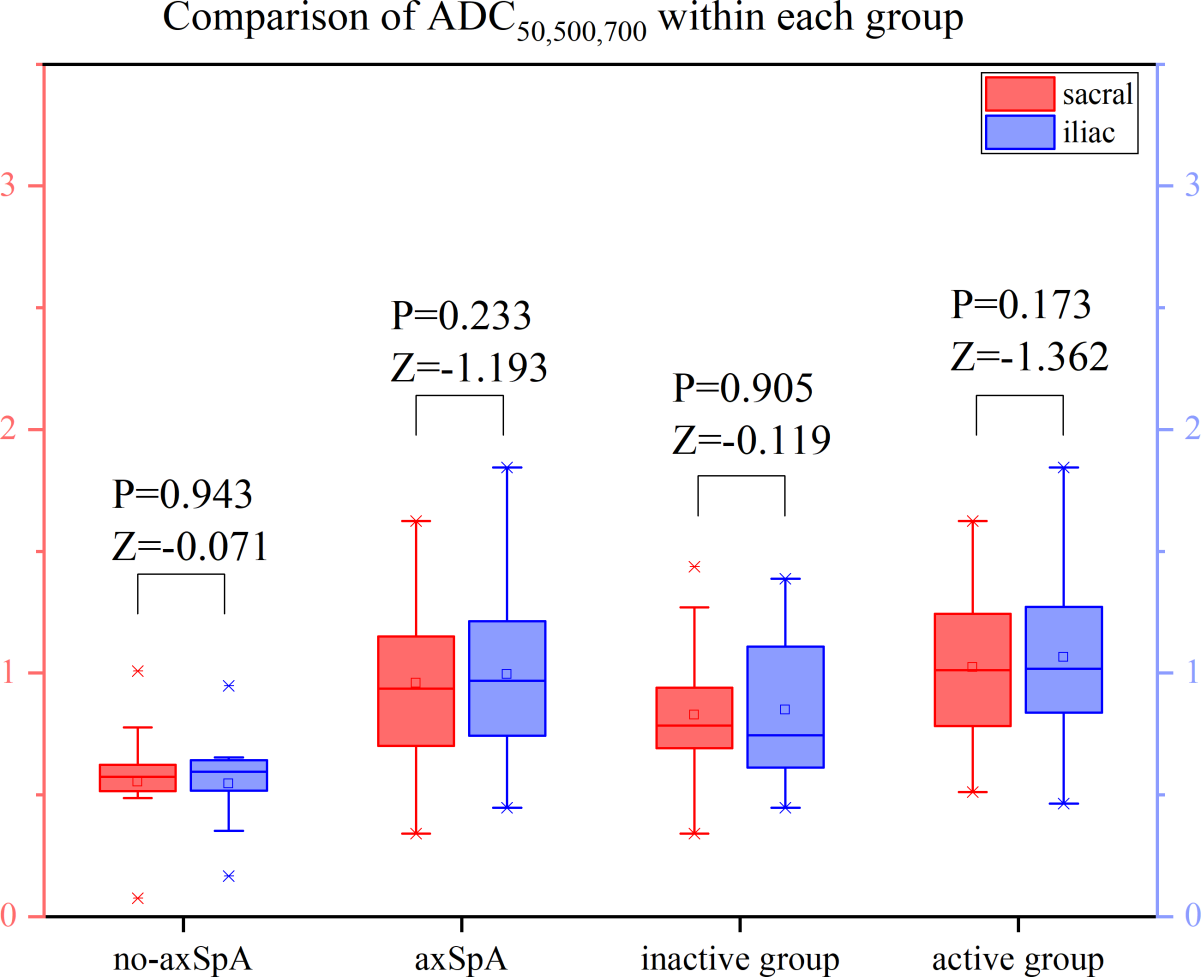
Boxplot for Comparison of ADC_50,500,700_ within each group. p-value of <0.05 was considered statistically signifificant.

It was tested that the ADC values of the sacral and iliac side in the no-axSpA, axSpA, inactive group and active group were not normally distributed, and there was no significant difference in sacral and iliac ADC values in the four groups (all P>0.05). With the increase of b value, the ADC values within each group decreased in different degrees. For the most part ADC_50, 500,700_ values were lower than the values of ADC_50, 500_ and ADC_50,700_ (**Figure 6**–**8**, **Supplementary Table S2**).

### Comparison of different ADC values of sacroiliac joints between each group

The ADC values of axSpA, active and inactive group were higher than those of no-axSpA group, while those of active group were all higher than those of inactive group. The ADC values obtained by fitting with different b values were significantly different between no-axSpA and axSpA, no-axSpA and inactive, no-axSpA and active, inactive and active groups, no matter on the sacral side or the iliac side (P<0.05) (**Table 3**).

**Table 3:** Comparison of different ADC values between each group.

	no-axSpA vs axSpA		no axSpA vs inactive		no axSpA vs active		inactive vs active	
	H	P	H	P	H	P	H	P
sacral								
ADC_50,500_	16.901	<0.001	7.645	0.006	19.116	<0.001	7.364	0.007
ADC_50,700_	19.851	<0.001	9.369	0.002	22.125	<0.001	6.489	0.011
ADC_50,500,700_	25.672	<0.001	14.704	<0.001	26.607	<0.001	7.896	0.005
iliac								
ADC_50,500_	20.236	<0.001	6.555	0.010	25.354	<0.001	8.244	0.004
ADC_50,700_	20.082	<0.001	5.985	0.014	25.727	<0.001	6.943	0.008
ADC_50,500,700_	26.64	<0.001	10.664	0.001	31.363	<0.001	8.963	0.003

### Diagnostic efficacy of ADC values obtained by fitting with different b values between each group

According to the analysis of ROC curve, the AUCs of ADC_50, 500,700_ between no-axSpA and axSpA, no-axSpA and inactive, no-axSpA and active and inactive and active groups were higher than those of ADC_50,700_, ADC_50,500_ (**Table 4**, **Supplementary Figure S2-3**). The sensitivity of ADC_50, 500,700_ was better than ADC_50,700_ in most inter-group comparisons. The specificity of ADC_50, 500,700_ was better than ADC_50,500_ in most inter-group comparisons.

**Table 4:** Comparison of diagnostic efficacy of ADC values between each group.

		AUC	cut-off value	sensitivity	specificity	Youden index	P
no axSpA vs axSpA							
sacral	ADC_50,500_	0.816	0.757	0.843	0.706	0.549	<0.001
	ADC_50,700_	0.842	0.821	0.663	0.941	0.604	<0.001
	ADC_50,500,700_	0.889	0.636	0.854	0.824	0.678	<0.001
iliac	ADC_50,500_	0.845	0.789	0.831	0.824	0.655	<0.001
	ADC_50,700_	0.844	0.710	0.775	0.824	0.599	<0.001
	ADC_50,500,700_	0.896	0.659	0.831	0.941	0.772	<0.001
no-axSpA vs inactive							
sacral	ADC_50,500_	0.746	0.683	0.931	0.588	0.519	0.006
	ADC_50,700_	0.773	0.821	0.552	0.941	0.493	0.002
	ADC_50,500,700_	0.842	0.636	0.828	0.824	0.652	<0.001
iliac	ADC_50,500_	0.728	0.790	0.655	0.824	0.479	0.010
	ADC_50,700_	0.718	0.674	0.655	0.765	0.420	0.014
	ADC_50,500,700_	0.791	0.659	0.621	0.941	0.562	0.001
no axSpA vs active							
sacral	ADC_50,500_	0.849	0.878	0.767	0.824	0.591	<0.001
	ADC_50,700_	0.875	0.823	0.717	0.941	0.658	<0.001
	ADC_50,500,700_	0.912	0.780	0.767	0.941	0.708	<0.001
iliac	ADC_50,500_	0.902	0.852	0.867	0.882	0.749	<0.001
	ADC_50,700_	0.905	0.715	0.867	0.824	0.691	<0.001
	ADC_50,500,700_	0.947	0.665	0.933	0.941	0.874	<0.001
inactive vs active							
sacral	ADC_50,500_	0.678	1.063	0.583	0.793	0.376	0.007
	ADC_50,700_	0.667	1.089	0.417	0.897	0.314	0.011
	ADC_50,500,700_	0.684	0.956	0.600	0.793	0.393	0.005
iliac	ADC_50,500_	0.689	0.936	0.783	0.586	0.369	0.004
	ADC_50,700_	0.673	0.731	0.867	0.448	0.315	0.008
	ADC_50,500,700_	0.697	0.726	0.900	0.483	0.383	0.003

### Comparison of ADC values among the treatment group in different cycles

**Figure 9:**
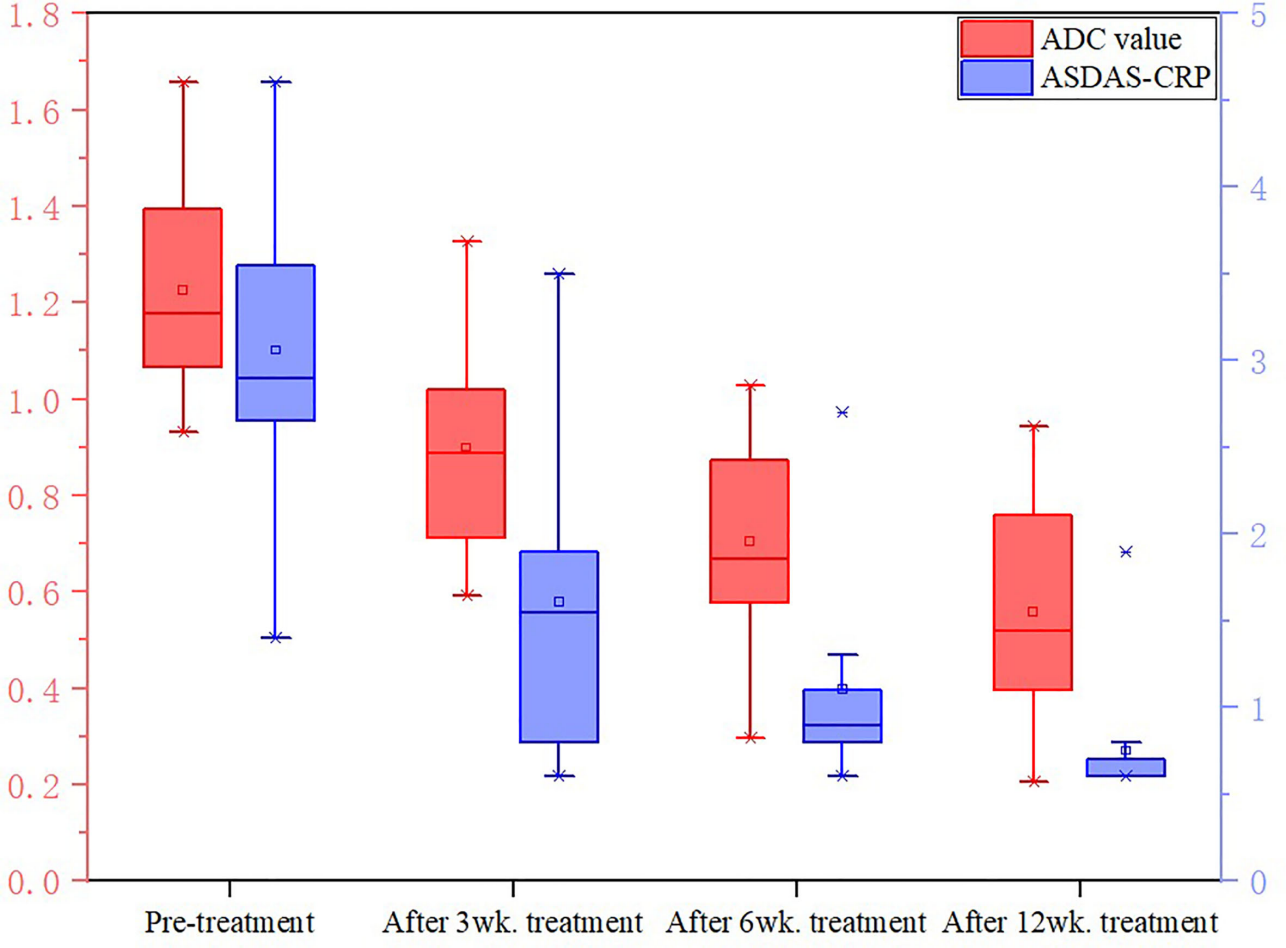
Changes in ADC values and ASDAS in the treatment group. The values of ADC and ASDAS-CRP in the treatment group decreased synchronously with the treatment period.

In the treatment group, the signal intensity of subchondral bone marrow decreased in different degrees in 3 weeks, 6 weeks and 12 weeks after treatment. PDWI-fs sequence showed that the signal intensity of subchondral bone marrow in sacroiliac joints decreased in varying degrees (**Figure 4A**_(1-4)_, **5A**_(1-4)_); DWI signal decreased in varying degrees (**Figure 4B**_(1-4)_, **5B**_(1-4)_); ADC value showed a downward trend (**Figure 4C**_(1-4)_, **5C**_(1-4)_); ADC values decreased with ASDAS-CRP values, synchronously (**Figure 9**). The median ADC values of the treatment group before treatment, 3 weeks, 6 weeks and 12 weeks after treatment were 1.177×10^-3^ mm^2^/s, 0.886×10^-3^ mm^2^/s, 0.668 ×10^-3^ mm^2^/s and 0.519 ×10^-3^ mm^2^/s, respectively.

Friedman test showed that there was significant difference in ASDAS-CRP between treatment groups(P<0.0083, Bonferroni-corrected threshold), except between the 3-week and 6-week treatment groups (P=0.141, Bonferroni-corrected threshold) and between the 6-week and 12-week treatment groups(P=0.027, Bonferroni-corrected threshold).

Friedman test showed that there was significant difference in ADC values between pre-treatment and 6-week treatment (P<0.0083, Bonferroni-corrected threshold), between pre-treatment and 12-week treatment (P<0.0083, Bonferroni-corrected threshold), and between 3-week treatment and 12-week treatment (P<0.0083, Bonferroni-corrected threshold). There was no significant difference in ADC values between pre-treatment and 3 weeks (P=0.086, Bonferroni-corrected threshold), 3 weeks and 6 weeks (P=0.072, Bonferroni-corrected threshold), 6 weeks and 12 weeks (P= 0.086, 0.072, 0.120, respectively. all P>0.0083, Bonferroni-corrected threshold).

## Discussion

The study verified that RESOLVE DWI technique can assist in diagnosis of diseases by showing the diffusion and distribution of water molecules in the microstructure of sacroiliac joints. Increased ADC values can be used to quantitatively evaluate subchondral BME of sacroiliac joints (11–13).

The b value and ADC value are two important parameters in DWI sequence. The b value affects the sensitivity of diffusion motion of water molecules and can reflect the degree of diffusion weighting. Theoretically, two different b values can be used to obtain the ADC graph and calculate the ADC value, which can reflect the dispersion movement and microcirculation perfusion status of water molecules in local tissues through objective quantitative values. Consequently, the selection of b value directly affects the accuracy of ADC value. Different from the IVIM study using the double-index model with multiple B-values, the influence of microcirculation perfusion in the single-index model (DWI) cannot be ignored (29). To reduce the influence of microcirculation perfusion, the common way is to use a high b value. The images with high b value increase the significance of lesions and improve the detection of lesions by reducing the influence of microcirculation perfusion, while the images quality may also be decreased by magnetic susceptibility artifacts and the reduction in signal-to-noise ratio (SNR) (30). In the several study, the commonly range of b values in sacroiliac joints scanning sequence were 500–800s/mm^2^ (13, 18, 31, 32), therefore, the b values used in our study were 50,500,700s/mm^2^.

This study displayed that the ADC values of sacroiliac joints in inactive group and active group were significantly higher than those in no-axSpA group, which was consistent with the results of Wang et al. (31), illustrating that subchondral BME was a meaningful sign in diagnosis of axSpA. Furthermore, BME was still present in subchondral bone marrow of inactive patients, indicating that though patients in the inactive stage, according to ASDAS, still cannot rule out inflammatory activity in sacroiliac joints, which is also the limitation of clinical subjective score. Compared with the inactive group, the ADC_50,500,700_ and ADC_50,700_ values of sacroiliac joints in the active group increased, displaying a higher signal on RESOLVE DWI images, indicating that with the aggravation of the disease, the degree of subchondral BME was further aggravated, which was consistent with the results of Zhang et al. (18).

The diagnostic efficiency of ADC values obtained by fitting with different b values were evaluated comprehensively according to ROC curve. In this study, the RESOLVE DWI images corresponding to ADC_50,500_ could clearly show the sacroiliac joints with good contrast, but the specificity to assess inflammation was decreased. Mostly due to the influence of microcirculation perfusion, ADC_50,500_ values were on the relatively high side, increasing the number of false positives. The RESOLVE DWI images corresponding to ADC_50,700_ were more accurate to assess inflammation, but the images were prone to deformation and artifact, and the image contrast decreased, resulting in inaccurate assessments of some inflammation and increasing the number of false negatives. Consequently, the sensitivity of ADC_50,700_ was inferior to ADC_50,500._ Meanwhile, it also explains the reason why the AUCs of ADC_50,500_ and ADC_50,700_ were similar. Furthermore, the AUC of ADC_50, 500,700_ was higher than those of ADC_50,500_ and ADC_50,700_. The results indicated that the diagnostic efficiency of ADC values obtained by multi-b value fitting were higher than those obtained by two b value fitting. We speculated that the background noise was effectively reduced in ADC_50,500,700_, and the higher image quality in ADC_50,500,700_ may preserve the higher ADC values in BME and prevented decreased ADC values from averaging with normal bone marrow.

Therefore, ADC_50,500,700_ values may be more reliable for diffusion quantification in BME than the values of ADC_50,500_ andADC_50,700_. As a consequence, the RESOLVE DWI images corresponding to ADC_50,500,700_ had relative higher sensitivity and specificity to assess inflammation, while taking into account the image quality. The results of the study manifested the detection of lesions depended not only on ADC values close to the real dispersion, correlated with the highest b value, but also on the clear anatomical structure, correlated with the low b value and the number of b values. ADC_50, 500,700_ is superior to ADC_50, 500_ and ADC_50,700_ in terms of overall image quality and diagnostic confidence, which can further improve the diagnosis of disease activity and help to optimize image quality.

In this study, it exhibited that after TNFi treatment, the ADC values in the treatment group were significantly lower than that before treatment, and ADC values decreased more in the treatment group with the prolongation of the treatment cycles, showing that the degree of BME decreased with the prolongation of the treatment cycles, which further proved that TNFi can effectively control the active state of axSpA disease, by inhibiting the abnormal immune response and inflammatory process mediated by TNF-α.

In the course of the study, it was worth noting that there was significant difference between groups with a difference of 6 weeks or more, but there was no significant difference when the interval was 3 weeks, indicating that the ADC values of BME decreased gradually and the short-term difference was not significant. Compared with the clinical symptoms and laboratory indexes (ASDAS-CRP) which were significantly improved in the short term after treatment, the dissipation of BME was relatively delayed. BME was one of the results of inflammatory reaction, mediated by inflammatory cells and inflammatory factors. Although TNFi inhibited part of the inflammatory process, it would take some time for BME to dissipate. Consequently, there were two recommendations of the study. Firstly, frequent reexamination in the early stage of treatment (less than 3 weeks or so) did not have higher benefits. Secondly, re-examination at 6 weeks or beyond without significant reduction of the ADC values of BME shows the inflammation of the sacroiliac joints is slow to resolve and the treatment plan should be adjusted clinically. The relationship between specific treatment period and reexamination needs to be further studied.

## Limitation

This study had some limitations. Firstly, it was a prospective study with a relatively small sample, which may cause the deviation of the results. Larger samples, multicenter, and randomized controlled trials is warranted. Secondly, although patients were divided based on ASDAS-CRP score, they were not subdivided into subgroups. The number and range of b values combinations is small. Increasing the grouping for further study may compensate for this disadvantage. Thirdly, in this study, the follow-up time of the patients in the treatment group was short, and no relapsing patients were found after remission. it is necessary to extend the follow-up time. Fourthly, because of the different treatment schemes and economic constraints of patients, patients who did not receive TNFi treatment were not followed up. It is the next step to evaluate the correlation between different treatment schemes and the prognosis of axSpA patients by imaging techniques.

## Conclusion

In conclusion, multi-b-values fitting (b=50,500,700s/mm^2^) RESOLVE DWI provided an effective quantitative indicator for evaluating the inflammatory activity of sacroiliac joints and the curative effect of TNFi in axSpA. It was worth noting that short-term review (3 weeks or less) of RESOLVE DWI was unsatisfactory and review at 6 weeks or later would help to evaluate the curative effect of axSpA.

## Data availability statement

The original contributions presented in the study are included in the article/**Supplementary Material**. Further inquiries can be directed to the corresponding author.

## Ethics statement

The studies involving human participants were reviewed and approved by the Ethics Committee of Fujian Province Hospital. Written informed consent to participate in this study was provided by the participants’ legal guardian/next of kin.

## Author contributions

XC and ML: designing the study and writing the manuscript. SSY: data collection and data analysis. FG and MM gave advice in the statistical analysis and data interpretation. SY: revise the manuscript. All authors contributed to the article and approved the submitted version.
