# Supplementary material for: Multi-b-values-fitting readout-segmentation of long variable echo-trains diffusion-weighted imaging (RESOLVE DWI) in evaluation of disease activity and curative effect of axial spondyloarthritis (axSpA)
Source: Front Immunol. 2023 Jul 3;14:1136925. doi: 10.3389/fimmu.2023.1136925 (PMC10351283; doi:10.3389/fimmu.2023.1136925)
Supplement: Supplementary file 1 [file DataSheet_1.docx]

**Supplementary Figure Legends**

Figure S1, Flowchart of the study. ASAS, Assessment of Spondyloarthritis international Society; axSpA, axial spondyloarthritis; TNFi, tumour necrosis factor inhibitors.

Figure S2, the receiver operating characteristic curves calculated from sacral ADC_50,500_, ADC_50,700_ and ADC_50,500,700_ to differentiate each group. (A) between no-axSpA and axSpA. (B) between no-axSpA and inactive group. (C) between no-axSpA and active group. (D) between inaxSpA and active group.

Figure S3, the receiver operating characteristic curves calculated from iliac ADC_50,500_, ADC_50,700_ and ADC_50,500,700_ to differentiate each group. (A) between no-axSpA and axSpA. (B) between no-axSpA and inactive group. (C) between no-axSpA and active group. (D) between inaxSpA and active group.

**Supplementary Tables**

**Table S1 Comparison of age and sex among each group**

|  | no-axSpA vs axSpA | no-axSpA vs Inactive | no-axSpA vs Active | Inactive vs Active |
| --- | --- | --- | --- | --- |
| Age |  |  |  |  |
| Z/H | -0.866(Z)^a^ | 1.010(H)^b^ | | |
| P | 0.386 | 0.603 | | |
| Sex |  |  |  |  |
| P^c^ | 0.886 | 0.989 | 0.832 | 0.783 |

a, Mann-Whitney U text

b, Kruskal Wallis text

c, Pearson's chi squared test

**Table S2 Comparison of different ADC values within each group (×10^-3^ mm^2^/s)**

|  | ADC_50,500_ | ADC_50,700_ | ADC_50,500,700_ |
| --- | --- | --- | --- |
| no-axSpA |  |  |  |
| sacral | 0.653(0.588,0.856) | 0.652(0.530,0.755) | 0.574(0.502,0.629) |
| iliac | 0.679(0.598,0.774) | 0.604(0.560,0.682) | 0.596(0.487,0.644) |
| Z | -0.166 | -0.26 | -0.071 |
| P | 0.868 | 0.795 | 0.943 |
| axSpA |  |  |  |
| sacral | 1.047(0.814,1.310) | 0.954(0.731,1.164) | 0.935(0.701,1.170) |
| iliac | 1.105(0.850,1.303) | 0.903(0.739,1.237) | 0.969(0.743,1.216) |
| Z | -0.878 | -0.972 | -1.193 |
| P | 0.380 | 0.331 | 0.233 |
| inactive group |  |  |  |
| sacral | 0.885(0.760,1.056) | 0.832(0.677,1.022) | 0.783(0.686,0.944) |
| iliac | 0.874(0.736,1.266) | 0.811(0.615,1.186) | 0.744(0.610,1.161) |
| Z | -0.335 | -0.054 | -0.119 |
| P | 0.738 | 0.957 | 0.905 |
| active group |  |  |  |
| sacral | 1.147(0.885,1.360) | 1.000(0.778,1.294) | 1.012(0.783,1.248) |
| iliac | 1.136(0.948,1.338) | 1.037(0.793,1.274) | 1.017(0.836,1.281) |
| Z | -1.281 | -1.332 | -1.362 |
| P | 0.200 | 0.183 | 0.173 |
