# Supplementary figures and images for: Multi-b-values-fitting readout-segmentation of long variable echo-trains diffusion-weighted imaging (RESOLVE DWI) in evaluation of disease activity and curative effect of axial spondyloarthritis (axSpA)
Source: Front Immunol. 2023 Jul 3;14:1136925. doi: 10.3389/fimmu.2023.1136925 (PMC10351283; doi:10.3389/fimmu.2023.1136925)

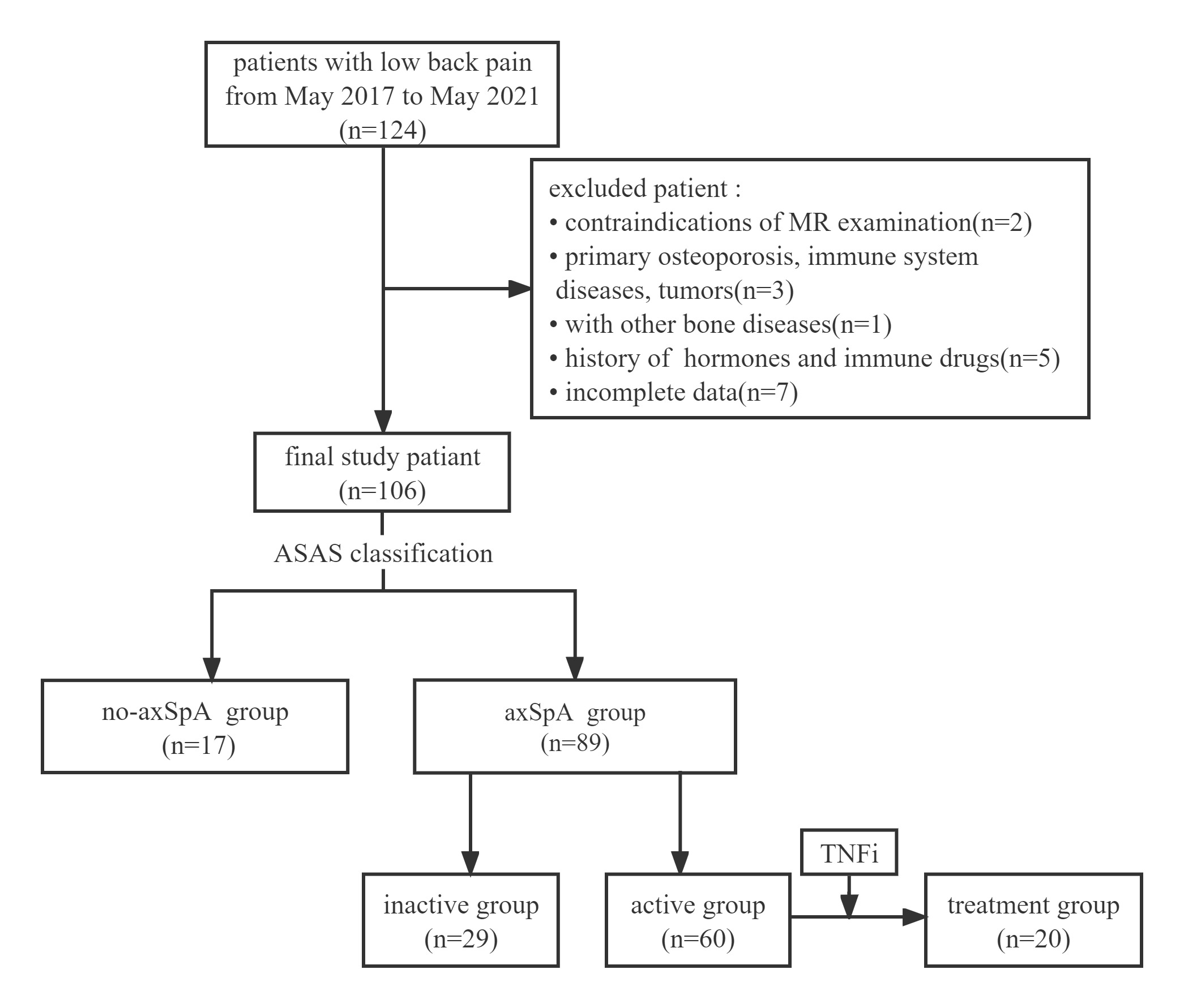

Supplement: Supplementary file 2 [file Image_1.tif]

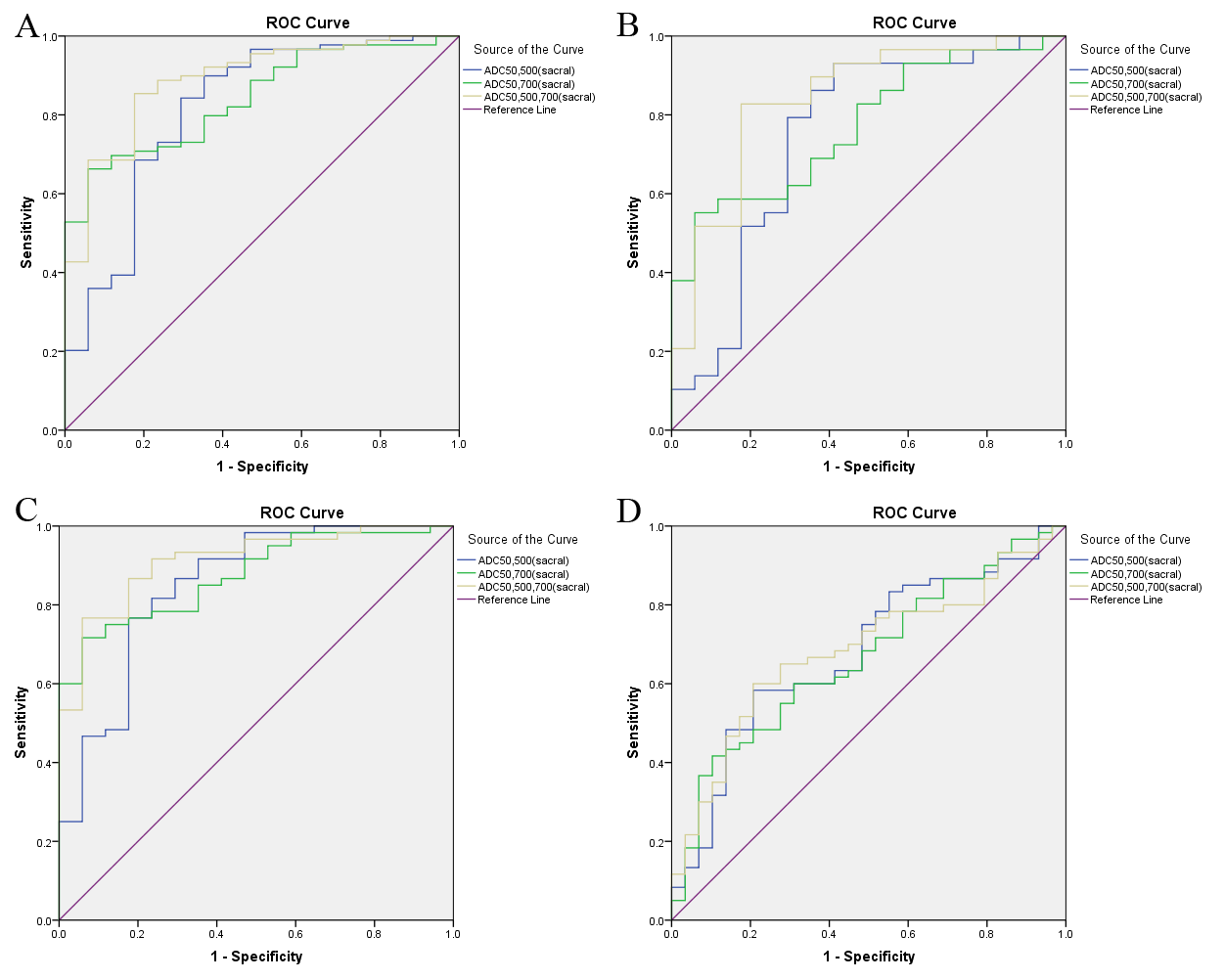

Supplement: Supplementary file 3 [file Image_2.tif]

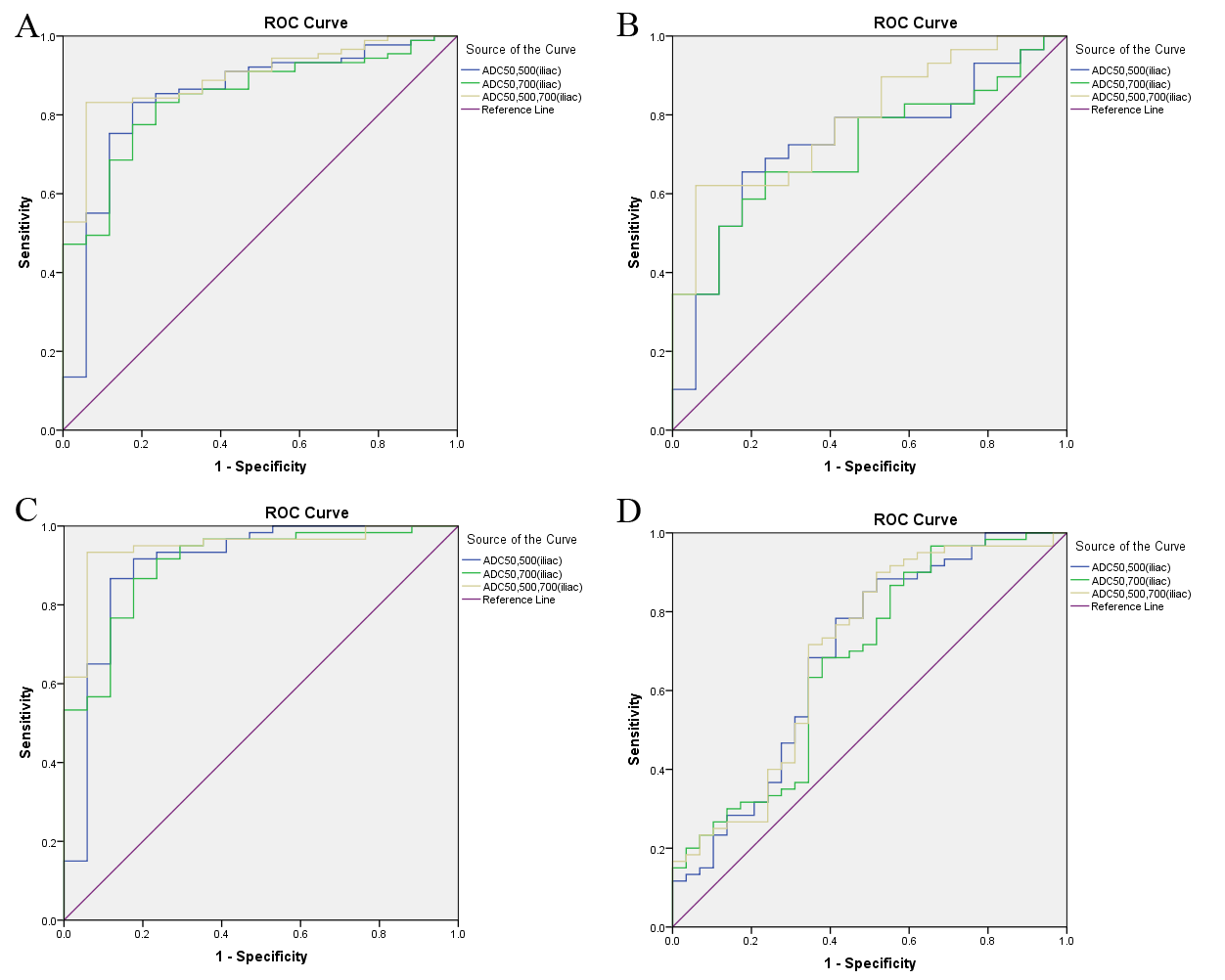

Supplement: Supplementary file 4 [file Image_3.tif]
